# Therapeutic Effects of BCG Vaccination on Type 1 Diabetes Mellitus: A Systematic Review and Meta-Analysis of Randomized Controlled Trials

**DOI:** 10.1155/2020/8954125

**Published:** 2020-03-26

**Authors:** Yu-Chen Chang, Chien-Ju Lin, Yu-Hsuan Hsiao, Yu-Han Chang, Shu-Jung Liu, Hsin-Yin Hsu

**Affiliations:** ^1^Department of Family Medicine, MacKay Memorial Hospital, No. 92, Section 2, Zhongshan North Road, Taipei City 10449, Taiwan; ^2^Department of Family Medicine, Hsinchu MacKay Memorial Hospital, No. 690, Section 2, Guangfu Road, East District, Hsinchu City 30071, Taiwan; ^3^Department of Medical Library, MacKay Memorial Hospital, Tamsui Branch, No. 45, Minsheng Road, Tamsui District, New Taipei City 25160, Taiwan; ^4^Institute of Epidemiology and Preventive Medicine, National Taiwan University, No. 17, Xu-Zhou Rd., Taipei City 10055, Taiwan

## Abstract

**Background:**

Type 1 diabetes mellitus (T1DM) causes the irreversible destruction of pancreatic beta cells. The Bacillus Calmette–Guerin (BCG) vaccine can modulate the immune response and decelerate disease progression. The aim of this study is to investigate the efficacy of the BCG vaccine for the treatment of T1DM.

**Objective:**

Six databases were systematically searched from inception to the end of August 2019. The randomized controlled trials (RCTs) that evaluated glycemic control in response to the BCG vaccine for T1DM were enrolled. The primary outcome was glycated hemoglobin (HbA1c) level, and secondary outcomes included fasting and stimulated C-peptide level, daily insulin dosage, and clinical remission. The revised Cochrane risk of bias tool was used for quality assessment, and meta-analyses were conducted to evaluate the efficacy of the BCG vaccine.

**Results:**

Four studies with a total of 198 subjects were included. The results of HbA1c and fasting C-peptide levels were extracted for further quantitative assessment. The pooled meta-analysis demonstrated no significant difference in HbA1c levels (mean difference [MD], −0.12; 95% confidence interval [CI], −0.53 to 0.30; *I*^2^ = 56%) or fasting C-peptide levels (MD, −0.15; 95% CI, −0.35 to 0.06; *I*^2^ = 0%) in the BCG intervention group as compared with that in the placebo group.

**Conclusions:**

There is no robust evidence to support the use of the BCG vaccine for the treatment of T1DM although the HbA1c levels tended to improve. Additional RCTs to assess the long-term effects of the BCG vaccine on glycemic control are warranted.

## 1. Introduction

Type 1 diabetes mellitus (T1DM) involves the autoimmune destruction of pancreatic beta cells, which eventually leads to absolute insulin deficiency. Diet control with daily injections of insulin remains the standard treatment strategy. Despite mounting advances in disease care, the global incidence of T1DM reportedly increases by 2%–3% yearly, with the highest risk in European-derived populations [1, 2]. The incidence of T1DM has increased by approximately 70% among young children over the past 20 years [3]. Hypoglycemia and ketoacidosis are its life-threatening complications. Severe hypoglycemic events contribute to 4%–10% of disease-related deaths. Hospitalization for diabetic ketoacidosis accounts for 13%–19% of type 1 diabetes-related mortality [1]. Retinopathy, diabetic renal disease, neuropathy, and cardiovascular disease are the major causes of morbidity and mortality in subjects with T1DM. Cardiovascular disease results in 8–13-year shorter life expectancy for T1DM patients than for healthy subjects [1]. According to a population-based study conducted in the USA, the economic burden per case of T1DM surpassed that of type 2 diabetes, and the annual direct and indirect costs are predicted to exceed $14.4 billion [4].

Bacillus Calmette–Guerin (BCG) is one of the oldest vaccines in use. The World Health Organization recommends the BCG vaccine for all children born in countries with a high incidence of tuberculosis and/or leprosy [5]. Over the past few decades, the broad immunological effects of the BCG vaccine have been reported, which include the diversity of autoimmune, allergic, and induced adaptive immune responses to childhood infections [6]. In multiple sclerosis, BCG yields favorable effects with reduction of disease activity through potential modulation of T-cell-mediated autoimmunity [7]. Additionally, intravesical injection of BCG eradicates superficial bladder tumors by invoking a strong local immune reaction [8]. The BCG vaccine is thought to activate the reprogramming of immune cells and alter cellular metabolism, including the acceleration of glycolysis [9–11]. Several studies on nonobese diabetic (NOD) murine models have reported that the BCG vaccine initiates an immuno-modulation process that suppresses insulitis [12, 13]. Furthermore, a pilot trial by Shehadeh et al. found that a single injection of the BCG vaccine induced clinical remission in 65% of recent-onset T1DM patients as compared with 7% of controls [14].

Although accumulating evidence suggests a beneficial effect of the BCG vaccine against T1DM, most studies have employed the NOD mouse model of T1DM, while studies on human populations have remained scarce. In addition, there is a wide range of heterogeneity among reports. Therefore, the aim of this systematic review and meta-analyses was to investigate the treatment benefit of the BCG vaccine on glycemic control in T1DM patients.

## 2. Materials and Methods

### 2.1. Data Sources and Search Strategy

The review protocol has been registered in the PROSPERO International Prospective Register of Systematic Reviews (registration number: CRD42020152113) and was conducted in accordance with the preferred reporting items for systematic reviews and meta-analysis statements [15] ([Supplementary-material supplementary-material-1]). The Embase, PubMed, the Cochrane Central Register of Controlled Trials, the Cochrane Database of Systematic Reviews, Cumulative Index to Nursing and Allied Health, and PerioPath: Index to Taiwan Periodical Literature databases were systematically searched from the earliest date available to the end of August 2019 without any language or time restriction with the following medical subject headings combined by applying Boolean operators: “BCG vaccine,” “Bacillus Calmette Guerin,” “type 1 diabetes mellitus,” “insulin-dependent diabetes mellitus,” “glycated hemoglobin,” and “blood sugar.” A professional librarian (S.-J.L.) reviewed and appropriately organized the terms to improve the sensitivity and specificity of the search strategy. [Supplementary-material supplementary-material-1] shows the details of the search strategy.

### 2.2. Study Selection

Three authors (Y.-H.H., Y.-H.C., and C.-J.L.) independently screened the titles and abstracts of each study that met the inclusion criteria, and any controversy was resolved by consensus through discussion with the fourth author (Y.-C.C.). Two independent reviewers (C.-J.L. and Y.-C.C.) assessed the eligibility of each publication on the basis of the full text after the initial search. The eligibility criteria for included randomized control trials (RCT) were as follows: (1) studies on T1DM patients, regardless of age, age of disease onset, or disease duration; (2) one treatment group received the BCG vaccine with a control group; and (3) reporting of glycemic control as an outcome measure after the intervention. The following were excluded: (1) nonhuman studies, (2) studies reporting the prevention of new-onset diabetes as the main outcome, (3) duplicate publications, and (4) crossover studies. If multiple reports originated from the same study population, then the most complete or latest report was included.

### 2.3. Data Extraction

Two authors (Y.-H.H. and Y.-H.C.) independently extracted the data into predesigned customized spreadsheets, and any inconsistency was resolved by discussion with the third author (Y.-C.C.). The following information was retrieved: first author's name, year of publication, country of publication, numbers and ages of subjects, intervention design, BCG type, outcome measures (including the timing of assessment), and main findings. The primary outcome was glycated hemoglobin (HbA1c) level. HbA1c level could determine 3-month average blood sugar level and has been regarded as an essential parameter for judging the degree of glycemic control [16]. Other relevant surrogate markers for glucose management were also applied as our secondary outcomes: (1) fasting and stimulated C-peptide level, (2) daily insulin dosage, and (3) clinical remission rate. In accordance with previous research [14], clinical remission was defined as exogenous insulin requirements of less than 0.2 U/kg per day while achieving adequate glycemic control for at least 4 weeks. C-peptide can be used to detect the secretion of endogenous insulin and is regarded as an indicator of beta cell function [17]. If an incomplete report was noticed, then the corresponding author of the original article was contacted to request the missing data.

### 2.4. Quality Assessment of Included Studies

Two reviewers (Y.-H.H. and Y.-H.C.) independently assessed the risk of bias using the revised Cochrane risk of bias tool for randomized trials (RoB 2.0). This approach specifies three levels of quality (“high,” “some concerns,” and “low”) across five domains. The randomization process, deviations from the intended interventions (effect of assignment to intervention), missing outcome data, outcome measures, and selection of the reported results were assessed. In general, we conducted assessments at the study level. When the consideration was specifically associated with outcome measures (e.g., “outcome measures”), assessment was performed according to our primary outcome (HbA1c level). If HbA1c level was not obtained in the study, we employed the reported secondary outcomes. Any disagreement was resolved by discussions with the third author (Y.-C.C.).

### 2.5. Statistical Analyses

All the statistical analyses were conducted using RStudio software (version 1.2.1335; RStudio, Inc., Boston, MA, USA). A random effects model was selected for the overall estimation as it was impossible to determine the exact treatment effect in advance, and there was significant heterogeneity among the included studies [18]. The pooled estimates of the mean difference (MD) and 95% confidence interval (CI) were calculated. Statistical heterogeneity was assessed using the *I*^2^ statistic and Cochran's *Q*-test. A probability (*p*) value of <0.10 for the *χ*^2^ of the *Q* statistic or an *I*^2^ > 50% was considered indicative of statistically significant heterogeneity [19]. Metaregression analyses were performed to examine the influence of clinical variables as the possible origins of heterogeneity, such as the age of participants, disease duration, and BCG dosage. The sensitivity analysis by omitting each study was also performed to evaluate the statistical robustness of the results. Further publication bias and subgroup analyses were not possible due to the limited number of selected studies.

## 3. Results

### 3.1. Search Results and Study Characteristics

**Figure 1:**
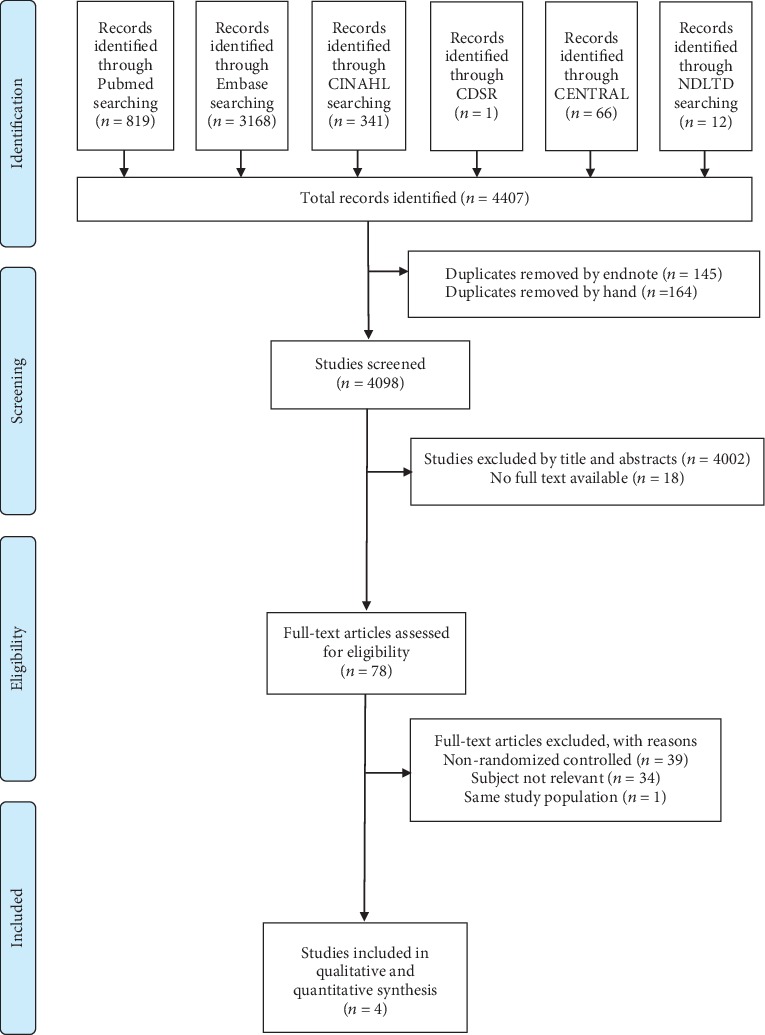
PRISMA study selection flow diagram. CINAH: Cumulative Index to Nursing and Allied Health; CDSR: Cochrane Database of Systematic Reviews; CENTRAL: Cochrane Central Register of Controlled Trials; NTLTD: Networked Digital Libraries of Theses and Dissertations.

In total, 4,407 potentially relevant publications were identified by the database search, of which 309 were duplicates and 4,002 were excluded after screening of the titles and abstracts (Figure 1); thus, 78 full-text articles were further assessed for eligibility. Finally, a total of four studies met the inclusion criteria and were included for qualitative synthesis and critical review [20–23].  Table 1 depicts the baseline characteristics of the included studies.

Of these studies, two were conducted in the USA [22, 23], one in Canada [21], and one in Italy [20]. Of the four included studies, three were conducted before 2000 [20–22], and one was published in 2018 [23]. The sample sizes ranged from 6 to 94, with a total of 198 subjects. The mean age of the examinees ranged from 10.1 to 36.0 years, and the follow-up duration ranged from 1 to 8 years. Three studies included participants with new-onset T1DM [20–22], whereas one study recruited cases with long-term diabetes (disease duration > 20 years) [23]. In addition, a single injection of the BCG vaccine was applied in the former three studies [20–22], while in the latter article, two injections of the BCG vaccine were administered 4 weeks apart [23]. All of the included studies compared the BCG intervention and control groups, and one study additionally incorporated a reference group of subjects with T1DM [23]. HbA1c was an outcome measure available in all studies. However, some raw outcome data of interest were unavailable across papers, including fasting and stimulating C-peptide levels, daily insulin dosages, and clinical remission rates.

### 3.2. Quality Assessments

[Supplementary-material supplementary-material-1] exhibits the results of the full qualitative assessments of all RCTs. In general, the included studies reported “some concerns” to “high” potential for bias, as demonstrated using the RoB 2.0 tool for randomized trials. One publication was rated “high risk” in the domain for missing outcome data because it did not report the precise number of missing participants and its effects on the outcome measures. We could not obtain information regarding potentially biased results nor the correlation between missingness of outcome and its true value [20]. One study was rated “some concerns” during the selection of the randomization process and reported result domains because it only mentioned that patients were randomized pairwise without providing the detailed allocation sequence. Further, the prespecified analysis plan was unavailable [21]. One study was rated “some concerns” because the prespecified analysis plan was noted presented [22]. Another study was also rated “some concerns” because significant baseline differences were present between the intervention and placebo groups in the randomization process domain (e.g., patient age, age at disease onset, and diabetes duration) [23].

### 3.3. Data Synthesis and Meta-Analyses

The treatment effect of the BCG vaccine on the glycemic control among subjects with T1DM was quantitatively assessed. Results of our primary outcome, HbA1c level, are available in three studies [20, 22, 23]. Results of fasting C-peptide levels, as one of our secondary outcome measures, are presented in two studies [20, 21]. However, the precise data for other secondary outcomes are incompletely reported across different studies. Stimulating C-peptide levels are reported in one study [21]. Daily insulin dosages and clinical remission rates are provided in another study [20]. Therefore, we only extracted results of HbA1c and fasting C-peptide levels for further meta-analyses.

### 3.4. Effects of the BCG Vaccine on HbA1c Levels

**Figure 2:**
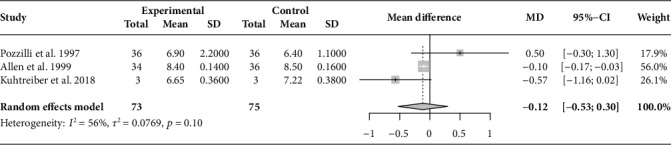
Forest plot of pooled HbA1c level (%) after the intervention (overall meta-analysis). HbA1c: glycated hemoglobin; MD: mean difference; CI: confidence interval.

In three of the selected studies [20, 22, 23], the HbA1c levels of 73 participants in the BCG intervention group tended to improve but not significantly (MD = −0.12, 95%CI = −0.53 to 0.30; *I*^2^ = 56%) as compared with that of the 75 participants in the placebo group (Figure 2). Among these studies, one reported a significantly positive effect of the BCG vaccine on HbA1c levels [23]. However, the sample size of this study was much smaller than that of the other two [20, 22]. The results remained unchanged after removing each study in the sensitivity analysis ([Supplementary-material supplementary-material-1]).

### 3.5. Effects of the BCG Vaccine on Fasting C-Peptide Levels

**Figure 3:**
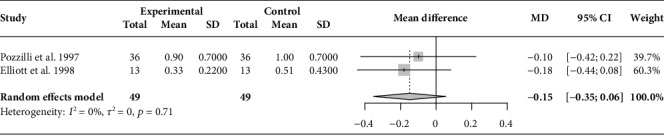
Forest plot of pooled fasting C-peptide level (ng/ml) after the intervention (overall meta-analysis). MD: mean difference; CI: confidence interval.

After pooling the results of two selected studies [20, 21] in the meta-analysis, there was no significant difference in the fasting C-peptide levels of 49 participants in the BCG intervention group (MD, −0.15; 95%CI = −0.35 to 0.06; *I*^2^ = 0%) as compared with that of the 49 participants in the placebo group (Figure 3).

Metaregression analysis was also performed to assess the effect of moderator variables on outcome measures among the selected studies. The results showed that participant age (*p* = 0.49), disease duration (*p* = 0.43), and BCG dosage (*p* = 0.38) had no effect on the efficacy of the BCG vaccine on T1DM.

## 4. Discussion

To our knowledge, this is the first systematic review and meta-analysis illustrating the therapeutic effects of the BCG vaccine for T1DM patients. The main results showed no statistical differences in HbA1c or fasting C-peptide levels after the intervention as compared with a placebo although HbA1c levels tended to improve following administration of the BCG vaccine. However, the combined effect size and related analysis should be interpreted with caution because of the small sample size and limited number of trials.

To date, T1DM remains an incurable autoimmune disease characterized by the progressive and irreversible destruction of pancreatic beta cells. In addition to traditional insulin replacement therapy, several efforts aiming at curing T1DM have focused on altering the autoimmune reaction. The approach began with trials of immunosuppressants, such as cyclosporin, and monoclonal antibodies [1]. Antigen-based therapies targeting the glutamate decarboxylase protein to induce immune tolerance have also been proposed [24, 25]. However, the high heterogeneity of the immune-mediated destruction process and large disparities between study designs hamper the development of effective interventions. The considerable side effects also compromise their broad use [25–28]. Cell therapies, such as islet transplantation, have been shown to promote *β*-cell neogenesis and proliferation in animal models, but only a minority of islet transplant recipients achieves persistent insulin independence [1]. Dietary approaches, such as vitamin D supplementation, have been shown to have the potential to modulate autoimmunity and improve glycemic control in T1DM patients with vitamin D deficiency in few studies [29, 30].

BCG possibly generates a suppressing effect through macrophages against a variety of lymphocyte functions [13]. Allen et al. found a significant decrease in islet cell antibody (ICA512bdc) levels in BCG-treated groups during a 2-year follow-up [22]. In another phase 1 trial preceding the study by Faustman et al., the increased amounts of regulatory T cells were observed in the BCG-treated subjects [31]. A preliminary trial by Shehadeh et al. of the administration of a single dose of the BCG vaccine in 17 newly diagnosed T1DM patients reported that clinical remission was achieved in 65% of subjects by week 4 and with a sustained effect in three cases for 6–10 months [14]. However, the sample size of this nonrandomized study was small, and most subsequent RCTs failed to reproduce similar efficacy [20–22]. The detailed mechanisms underlying the BCG vaccine remain unclear, and although the treatment benefits of the BCG vaccine could not be verified in the current analysis, there are several insights worth mentioning after comparing the disparities between studies.

### 4.1. BCG Dose, Strain, and Timing of Administration

In three negative studies, only a single injection of the BCG vaccine was administered [20–22]. By contrast, two doses of the BCG vaccine were given in the study by Kühtreiber et al. [23]. Although the standard regimen for the prevention of TB only requires a single dose of the BCG vaccine, the alteration of glycemic modulation and reprogramming of innate immune cells may necessitate a further booster dosage. Furthermore, the potency of different BCG strains may be inconsistent, as illustrated by the poor immune regulatory properties exhibited by the TICE strain of BCG [23]. Besides, in the study by Pozzilli et al., all of the subjects simultaneously received nicotinamide. Hence, the possibility of a masking effect by nicotinamide could not be excluded [20].

### 4.2. Follow-Up Duration

The follow-up duration of prior studies did not exceed 2 years [20–22]. However, even in the positive study by Kühtreiber et al., the reduction in HbA1c levels did not appear until three years after vaccination [23]. Notably, a similar onset time of the treatment effects of the BCG vaccine was also reported in another study of patients with multiple sclerosis [7]. The process of autoimmunity reversal may take more time to develop.

### 4.3. Characteristics of Study Subjects

All of the negative studies recruited patients with new-onset diabetes [20–22], whereas the positive study included adults with long-term T1DM [23]. The subjects in the latter study already had substantial losses of beta cells [32, 33]. The earlier hypothesis that the BCG vaccine could halt the destruction of pancreatic beta cells may less likely occur during this phase. None of the selected studies reported an improvement in C-peptide levels, a recognized marker of beta cell function. Allen et al. even found a trend toward a rapid decline in C-peptide values in the BCG group, which reached statistical significance for stimulated C-peptide levels in children [22]. On the other hand, Kühtreiber et al. reported an acceleration in glycolysis with a reduction in oxidative phosphorylation, which could more efficiently facilitate glucose utilization [23]. This novel finding indicated that the BCG vaccine may offer an additional effect beyond the preservation of beta cell function. However, in this study, subjects already received long-term medical therapy, and potential confounding factors such as their lifestyle and associated comorbidities were not mentioned.

We noticed a significant period of research among the selected papers. Three studies were published before the year 2000 [20–22], while only one trial was conducted afterward [23]. The multiple failures of prior trials may hinder advancements in this area. In addition, the progress of immunotherapy aimed at autoantibodies or activated T cells, which constitute the essential part of beta cell destruction, has redirected the attention of researchers in recent years.

The strength of this study is that it is the first systematic review and meta-analysis to explore available evidence of the BCG vaccine for the management of T1DM. This study also highlights the fact that knowledge in this field remains extremely limited to unveil the treatment effect of the BCG vaccine for T1DM patients. However, the BCG vaccine has been widely applied for decades for the prevention of TB with a coverage estimated as over 75% of the children born worldwide. At $2–3 per dose, BCG vaccination costs approximately $206 per year of healthy life gained, which is much less than the average annual income per person [34]. If a comprehensive mechanism and appropriate regimen can be established as evidence accumulates, owing to the relatively low expense, safety, and convenience, then the BCG vaccine is a cost-effective option to be incorporated routinely into the clinical management of T1DM. The combination with a complementary therapy such as vitamin D also merits further investigation.

There were several limitations to this study. First, the validity of our analysis was confined by a limited number of selective trials and small sample size. And it was impossible to perform additional subgroup analyses to investigate the variables contributing to the heterogeneity of the results. Second, the characteristics of the included studies varied, especially the demographics of the recruited patients, the vaccine type and regimen, and the follow-up duration. Thus, there was no consistent trend regarding the clinical value of the BCG vaccine. Third, the moderate to high risk of bias and insufficient outcome measures of the selected studies may also lessen the practicality of our results. Fourth, because most of the selected studies did not report major adverse effects, quantitative analysis to further verify the efficacy of the BCG vaccine could not be conducted. Lastly, the overall adverse reaction rate seemed higher in older people than in neonates in previous surveys, which is worth investigating in future research [35].

## 5. Conclusions

In summary, there is no robust evidence to support a significant benefit of the BCG vaccine for the treatment of T1DM. However, HbA1c levels tended to improve following administration. The results of quantitative analysis must be interpreted carefully due to the limited number of studies and small sample size. Additional RCTs are needed to enhance the body of evidence, evaluate the long-term effects of the BCG vaccine on glycemic control, as well as elucidate the underlying mechanisms.

## Figures and Tables

**Table 1:** Characteristics of randomized controlled trials investigating the effect of the BCG vaccine on T1DM.

Study	Country	Participants	Intervention	BCG type	Outcome measures	Main findings
Pozzilli et al. 1997 [20]	Italy	72 patients within 4 weeks of IDDM; mean age, 14.5 years	Intervention (*n* = 36): one dose of BCG with NCT 25 mg/kg; control (*n* = 36): NCT 25 mg/kg	Freeze-dried BCG vaccine (Berna Institute, base)	HbA1c, C-peptide, insulin dosage, and clinical remission; 1-year follow-up	No significant differences in insulin dosages or C-peptide (ng/ml) (0.8/0.9 vs. 0.93/1.0 preintervention/postintervention) and HbA1c (%) (9.7/6.9 vs. 9.8/6.4 preintervention/postintervention) level between BCG and control groups. BCG did not induce long-term clinical remission in IDDM patients.

Elliott et al. 1998 [21]	Canada	26 patients within 1 year of IDDM; mean age, 13.1 years	Intervention (*n* = 13): one dose of BCG; control (*n* = 13): placebo	BCG vaccine (Connaught, Toronto, Ontario, Canada)	HbA1c and fasting/stimulated C-peptide, and insulin dosage; 1.5-year follow-up	HbA1c, mean basal (ng/ml) (0.81/0.33 vs. 0.90/0.51 preintervention/postintervention), and stimulated (ng/ml) (1.35/0.51 vs. 1.74/0.84 preintervention/postintervention) C-peptide level, and insulin dosage did not differ significantly between BCG and control groups (*p* = 0.10–1.00).

Allen et al. 1999 [22]	U.S.	94 children with a diagnosis of new-onset IDDM; mean age, 10.5 years	Intervention (*n* = 47): one dose of BCG; control (*n* = 47): placebo	TICE BCG (PerImmune, Rockville, MD)	HbA1c, fasting/stimulated glucose and C-peptide, insulin dosage, and clinical remission; 2-year follow-up	No differences in HbA1c (%) (8.8/8.4 vs. 9.2/8.5 preintervention/postintervention), insulin requirement, and basal or stimulated C-peptide level between both groups. The trend toward a more rapid decrease in C-peptide level (*p* = 0.11) in the BCG group (*p* < 0.05).

Kühtreiber et al. 2018 [23]	U.S.	6 subjects with long-term diabetes; mean age, 42 years	Intervention (*n* = 3): two doses of BCG administered 4 weeks apart; control (*n* = 3): placebo	Lyophilized BCG (TheraCysH, Sanofi-Pasteur, Toronto, Ontario, Canada)	HbA1c and stimulated C-peptide; 8-year follow-up	After year 3, BCG lowered HbA1c (%) (7.36/6.65 vs. 7.1/7.22 preintervention/postintervention between intervention and control groups) to near normal levels for the next 5 years (*p* < 0.001 at year 8). No significant return of C-peptide level in the BCG group

BCG: Bacillus Calmette–Guerin; IDDM: insulin-dependent diabetes mellitus; NCT: nicotinamide; HbA1c: glycated hemoglobin.

## Data Availability

The data supporting this systematic review and meta-analysis are from previously reported studies and datasets, which have been cited. The processed data are available from the corresponding author upon reasonable request.

## Supplementary Materials

Supplementary Materials Table S1: PRISMA checklist. Table S2: Search strategy. Table S3: Revised Cochrane risk-of-bias tool (RoB 2.0) for quality assessment of included RCTs. Figure S1: Sensitivity analyses of BCG on pooled HbA1c level (%) in T1DM patients by omitting each study.
