# Supplementary material for: Therapeutic Effects of BCG Vaccination on Type 1 Diabetes Mellitus: A Systematic Review and Meta-Analysis of Randomized Controlled Trials
Source: J Diabetes Res. 2020 Mar 26;2020:8954125. doi: 10.1155/2020/8954125 (PMC7139880; doi:10.1155/2020/8954125)
Supplement: Supplementary Materials — Table S1: PRISMA checklist. Table S2: Search strategy. Table S3: Revised Cochrane risk-of-bias tool (RoB 2.0) for quality assessment of included RCTs. Figure S1: Sensitivity analyses of BCG on pooled HbA1c level (%) in T1DM patients by omitting each study. [file 8954125.f1.doc]

**Supplementary Materials**

**Table S1.** **PRISMA checklist.**

| Section/topic | # | Checklist item | Reported on page # |
| --- | --- | --- | --- |
| TITLE | | |  |
| Title | 1 | Identify the report as a systematic review, meta-analysis, or both. | 1 |
| ABSTRACT | | |  |
| Structured summary | 2 | Provide a structured summary including, as applicable: background; objectives; data sources; study eligibility criteria, participants, and interventions; study appraisal and synthesis methods; results; limitations; conclusions and implications of key findings; systematic review registration number. | 1-2 |
| INTRODUCTION | | |  |
| Rationale | 3 | Describe the rationale for the review in the context of what is already known. | 3 |
| Objectives | 4 | Provide an explicit statement of questions being addressed with reference to participants, interventions, comparisons, outcomes, and study design (PICOS). | 4 |
| METHODS | | |  |
| Protocol and registration | 5 | Indicate if a review protocol exists, if and where it can be accessed (e.g., Web address), and, if available, provide registration information including registration number. | 4 |
| Eligibility criteria | 6 | Specify study characteristics (e.g., PICOS, length of follow-up) and report characteristics (e.g., years considered, language, publication status) used as criteria for eligibility, giving rationale. | 5-6 |
| Information sources | 7 | Describe all information sources (e.g., databases with dates of coverage, contact with study authors to identify additional studies) in the search and date last searched. | 4-6 |
| Search | 8 | Present full electronic search strategy for at least one database, including any limits used, such that it could be repeated. | Table S2 |
| Study selection | 9 | State the process for selecting studies (i.e., screening, eligibility, included in systematic review, and, if applicable, included in the meta-analysis). | 5-6, Figure 1 |
| Data collection process | 10 | Describe method of data extraction from reports (e.g., piloted forms, independently, in duplicate) and any processes for obtaining and confirming data from investigators. | 5-6 |
| Data items | 11 | List and define all variables for which data were sought (e.g., PICOS, funding sources) and any assumptions and simplifications made. | 5-6 |
| Risk of bias in individual studies | 12 | Describe methods used for assessing risk of bias of individual studies (including specification of whether this was done at the study or outcome level), and how this information is to be used in any data synthesis. | 6 |
| Summary measures | 13 | State the principal summary measures (e.g., risk ratio, difference in means). | 6-7 |
| Synthesis of results | 14 | Describe the methods of handling data and combining results of studies, if done, including measures of consistency (e.g., I^2^) for each meta-analysis. | 6-7 |
| Risk of bias across studies | 15 | Specify any assessment of risk of bias that may affect the cumulative evidence (e.g., publication bias, selective reporting within studies). | 7 |
| Additional analyses | 16 | Describe methods of additional analyses (e.g., sensitivity or subgroup analyses, meta-regression), if done, indicating which were pre-specified. | 7 |
| RESULTS | | |  |
| Study selection | 17 | Give numbers of studies screened, assessed for eligibility, and included in the review, with reasons for exclusions at each stage, ideally with a flow diagram. | 7, Figure 1 |
| Study characteristics | 18 | For each study, present characteristics for which data were extracted (e.g., study size, PICOS, follow-up period) and provide the citations. | 9-11, Table 1 |
| Risk of bias within studies | 19 | Present data on risk of bias of each study and, if available, any outcome level assessment (see item 12). | 10-11, Table S3 |
| Results of individual studies | 20 | For all outcomes considered (benefits or harms), present, for each study: (a) simple summary data for each intervention group (b) effect estimates and confidence intervals, ideally with a forest plot. | 12-13, Figure 2, Figure 3 |
| Synthesis of results | 21 | Present results of each meta-analysis done, including confidence intervals and measures of consistency. | 11-12 |
| Risk of bias across studies | 22 | Present results of any assessment of risk of bias across studies (see Item 15). | NA |
| Additional analysis | 23 | Give results of additional analyses, if done (e.g., sensitivity or subgroup analyses, meta-regression [see Item 16]). | 12 |
| DISCUSSION | | |  |
| Summary of evidence | 24 | Summarize the main findings including the strength of evidence for each main outcome; consider their relevance to key groups (e.g., healthcare providers, users, and policy makers). | 12-16 |
| Limitations | 25 | Discuss limitations at study and outcome level (e.g., risk of bias), and at review-level (e.g., incomplete retrieval of identified research, reporting bias). | 16 |
| Conclusions | 26 | Provide a general interpretation of the results in the context of other evidence, and implications for future research. | 16 |
| FUNDING | | |  |
| Funding | 27 | Describe sources of funding for the systematic review and other support (e.g., supply of data); role of funders for the systematic review. | 17 |

**Table S2. Search strategy.**

Search strategy in Pubmed

| Search | Query |
| --- | --- |
| #26 | #8 AND #25 |
| #25 | #17 OR #24 |
| #24 | #22 AND #23 |
| #23 | Search (normal[Text Word] OR benign[Text Word] OR lower*[Text Word] OR decreas*[Text Word] OR reduc*[Text Word]) |
| #22 | #18 OR #19 OR #20 OR #21 |
| #21 | Search (Blood Glucose[Text Word] OR Blood Sugar[Text Word]) |
| #20 | Search (HbA1c[Text Word] OR HbA1[Text Word]) |
| #19 | Search Hemoglobin |
| #18 | Search Glycated Hemoglobin A |
| #17 | #9 OR #10 OR #11 OR #12 OR #13 OR #14 OR #15 OR #16 |
| #16 | Search Autoimmune Diabetes |
| #15 | Search (early onset diabetes[Text Word] OR ketoacidotic diabetes[Text Word] OR labile diabetes[Text Word]) |
| #14 | Search (IDDM[Text Word] OR T1D[Text Word] OR DM1[Text Word]) |
| #13 | Search type 1 diabet*[Text Word] |
| #12 | Search Brittle Diabetes[Text Word] |
| #11 | Search Insulin-Dependent Diabetes Mellitus 1 |
| #10 | Search Diabetes Mellitus Type I |
| #9 | Search Diabetes Mellitus, Type 1 |
| #8 | #1 OR #2 OR #3 OR #4 OR #5 OR #6 OR #7 |
| #7 | Search birth-vaccin*[Text Word] |
| #6 | Search (aeras 402[Text Word] OR aeras402[Text Word] OR mva 85a[Text Word] OR antigen 85A[Text Word] OR Ag85A[Text Word] OR eurocrine L3[Text Word] OR immucyst[Text Word] OR monovax[Text Word] OR mycobax[Text Word] OR onco tice[Text Word] OR oncotice[Text Word] OR pacis[Text Word] OR pastimmun[Text Word] OR theracys intravesical[Text Word]) |
| #5 | Search (Tubercul* Vaccin*[Text Word] OR antituberculosis vaccin*[Text Word] OR TB vaccin*[Text Word]) |
| #4 | Search (BCG vaccin*[Text Word] OR b.c.g. vaccin*[Text Word] OR Bacillus Calmette Guerin[Text Word] OR Calmette Vaccin*[Text Word]) |
| #3 | Search Bacterial Vaccines |
| #2 | Search Tuberculosis Vaccines |
| #1 | Search BCG Vaccine |

Search strategy in Embase

| Search | Query |
| --- | --- |
| #31 | #12 AND #30 |
| #30 | #23 OR #29 |
| #29 | #27 AND #28 |
| #28 | normal:ti,ab,kw OR benign:ti,ab,kw OR lower*:ti,ab,kw OR decreas*:ti,ab,kw OR oxidoreductase:ti,ab,kw |
| #27 | #24 OR #25 OR #26 |
| #26 | hba1c:ti,ab,kw OR hba1:ti,ab,kw OR 'blood glucose':ti,ab,kw OR 'blood sugar':ti,ab,kw |
| #25 | hemoglobin'/exp |
| #24 | glycosylated hemoglobin'/exp |
| #23 | #13 OR #14 OR #15 OR #16 OR #17 OR #18 OR #19 OR #20 OR #21 OR #22 |
| #22 | autoimmune disease'/exp |
| #21 | iddm:ti,ab,kw OR t1d:ti,ab,kw OR dm1:ti,ab,kw |
| #20 | labile NEAR/3 diabetes |
| #19 | ketoacidotic NEAR/3 diabetes |
| #18 | early NEAR/3 onset NEAR/3 diabetes |
| #17 | type NEAR/5 1 NEAR/5 diabet* |
| #16 | brittle NEAR/3 diabetes |
| #15 | diabetes AND mellitus AND type AND i |
| #14 | diabetes AND mellitus AND type AND 1 |
| #13 | insulin dependent diabetes mellitus'/exp |
| #12 | #1 OR #2 OR #3 OR #4 OR #5 OR #6 OR #7 OR #8 OR #9 OR #10 OR #11 |
| #11 | antigen 85a' OR ag85a |
| #10 | bacterial vaccine'/exp2019-06-04 |
| #9 | tb NEAR/3 vaccin*2019-06-04 |
| #8 | tubercul* NEAR/3 vaccin* |
| #7 | theracys NEAR/5 intravesical |
| #6 | calmette NEAR/5 vaccine |
| #5 | bacill* NEAR/3 calmette NEAR/3 guerin NEAR/3 vaccine |
| #4 | b.c.g. NEAR/3 vaccin* |
| #3 | antituberculosis NEAR/3 vaccine |
| #2 | aeras 402' OR aeras402 OR immucyst OR monovax OR 'mva 85a' OR mva85a OR mycobax OR 'onco tice' OR oncotice OR pacis OR pastimmun |
| #1 | bcg vaccine'/exp |

Search strategy in Cochrane Library

|  | Query |
| --- | --- |
| #1 | MeSH descriptor: [BCG Vaccine] explode all trees |
| #2 | MeSH descriptor: [Tuberculosis Vaccines] explode all trees |
| #3 | MeSH descriptor: [Bacterial Vaccines] explode all trees |
| #4 | (BCG vaccine* OR b.c.g. vaccine* OR Bacillus Calmette Guerin OR bacill* Calmette-Guerin OR Calmette Vaccin* OR Tubercul* Vaccin* OR antituberculosis vaccine OR TB vaccine OR aeras 402 OR aeras402 OR mva 85a OR antigen 85A OR Ag85A OR eurocrine L3 OR immucyst OR monovax OR mycobax OR onco tice OR oncotice OR pacis OR pastimmun OR theracys intravesical birth-vaccinated OR bacterial) |
| #5 | #1 OR #2 OR #3 OR #4 |
| #6 | MeSH descriptor: [Diabetes Mellitus, Type 1] explode all trees |
| #7 | MeSH descriptor: [Autoimmune Disease] explode all trees |
| #8 | (Diabetes Mellitus Type 1 OR Diabetes Mellitus Type I OR Insulin-Dependent Diabetes Mellitus 1 OR Brittle Diabetes OR type 1 diabet* OR Autoimmune Diabetes OR IDDM OR T1D OR DM1 OR early onset diabetes OR ketoacidotic diabetes OR labile diabetes):ti, ab, kw |
| #9 | #6 OR #7 OR #8 |
| #10 | MeSH descriptor: [Glycated Hemoglobin A] explode all trees |
| #11 | MeSH descriptor: [Hemoglobins] explode all trees |
| #12 | (HbA1C OR HbA1 OR Blood Glucose OR Blood sugar):ti, ab,kw |
| #13 | #10 OR #11 OR #12 |
| #14 | (normal OR benign OR lower* OR decreas* OR reduc*):ti, ab,kw |
| #15 | #13 AND #14 |
| #16 | #9 OR #15 |
| #17 | #5 AND #16 |

Search strategy in CINAHL

| Search ID# | Search Terms | Search Options |
| --- | --- | --- |
| S17 | S5 AND S16 | Search modes - Find all my search terms |
| S16 | S9 OR S15 | Search modes - Find all my search terms |
| S15 | S13 AND S14 | Search modes - Find all my search terms |
| S14 | TIAB normal OR benign OR lower* OR decreas* | Search modes - Find all my search terms |
| S13 | S10 OR S11 OR S12 | Search modes - Find all my search terms |
| S12 | TIAB Glycated Hemoglobin A OR Hemoglobin OR HbA1C OR HbA1 OR Blood Glucose OR Blood sugar | Search modes - Find all my search terms |
| S11 | (MH "Hemoglobins") | Search modes - Find all my search terms |
| S10 | (MH "Hemoglobin A, Glycosylated") OR (MH "Hemoglobin A") | Search modes - Find all my search terms |
| S9 | S6 OR S7 OR S8 | Search modes - Find all my search terms |
| S8 | TIAB Diabetes Mellitus Type 1 OR Insulin-Dependent Diabetes Mellitus 1 OR Brittle Diabetes Mellitus OR Type 1 Diabetes OR type 1 diabetic OR Autoimmune Diabetes OR IDDM OR T1D | Search modes - Find all my search terms |
| S7 | (MH "Autoimmune Diseases") | Search modes - Find all my search terms |
| S6 | (MH "Diabetes Mellitus, Type 1") | Search modes - Find all my search terms |
| S5 | (S1 OR S2 OR S3 OR S4) | Search modes - Find all my search terms |
| S4 | TX BCG vaccine* OR Bacillus Calmette Guerin OR bacill* Calmette‐Guerin OR Calmette Vaccine OR Tuberculosis Vaccine* OR TB vaccine OR birth-vaccinated OR vaccines | Search modes - Find all my search terms |
| S3 | (MH "Vaccines") | Search modes - Find all my search terms |
| S2 | (MH "Bacterial Vaccines") | Search modes - Find all my search terms |
| S1 | (MH "BCG Vaccine") | Search modes - Find all my search terms |

Search strategy in NDLTD

| Search ID# | Query |
| --- | --- |
| #1 | (卡介苗 + BCG + 疫苗) * (糖尿 + diabet*) |

**Table S3. Revised Cochrane risk-of-bias tool (RoB 2.0) for quality assessment of included RCTs**

| Domain1 | Risk of bias arising from the randomization process | |
| --- | --- | --- |
| Domain2 | Risk of bias due to deviations from the intended interventions | |
| Domain3 | Risk of bias due to missing outcome data | |
| Domain4 | Risk of bias in measurement of the outcome | |
| Domain5 | Risk of bias in selection of the reported result | |
| Overall risk of bias | Low risk of bias | The study is judged to be at low risk of bias for all domains for this result. |
|  | Some concerns | The study is judged to raise some concerns in at least one domain for this result, but not to be at high risk of bias for any domain. |
|  | High risk of bias | The study is judged to be at high risk of bias in at least one domain for this result. Or  The study is judged to have some concerns for multiple domains in a way that substantially lowers confidence in the result. |

|  | Domain 1 | Domain 2 | Domain 3 | Domain 4 | Domain 5 | Overall risk |
| --- | --- | --- | --- | --- | --- | --- |
| Kühtreiber et al. 2018 | Some concerns^a^ | Low | Low | Low | Low | Some concerns |
| Allen et al. 1999 | Low | Low | Low | Low | Some concerns^b^ | Some concerns |
| Elliott et al. 1998 | Some concerns^c^ | Low | Low | Low | Some concerns^b^ | Some concerns |
| Pozzilli et al. 1997 | Some concerns^c^ | Low | High^d^ | Low | Some concerns^b^ | High |

^a^Imbalance in baseline difference ^b^Pre-specified analysis plan not mentioned. ^c^Allocation sequence not mentioned. ^d^ Results likely biased by missing outcome data


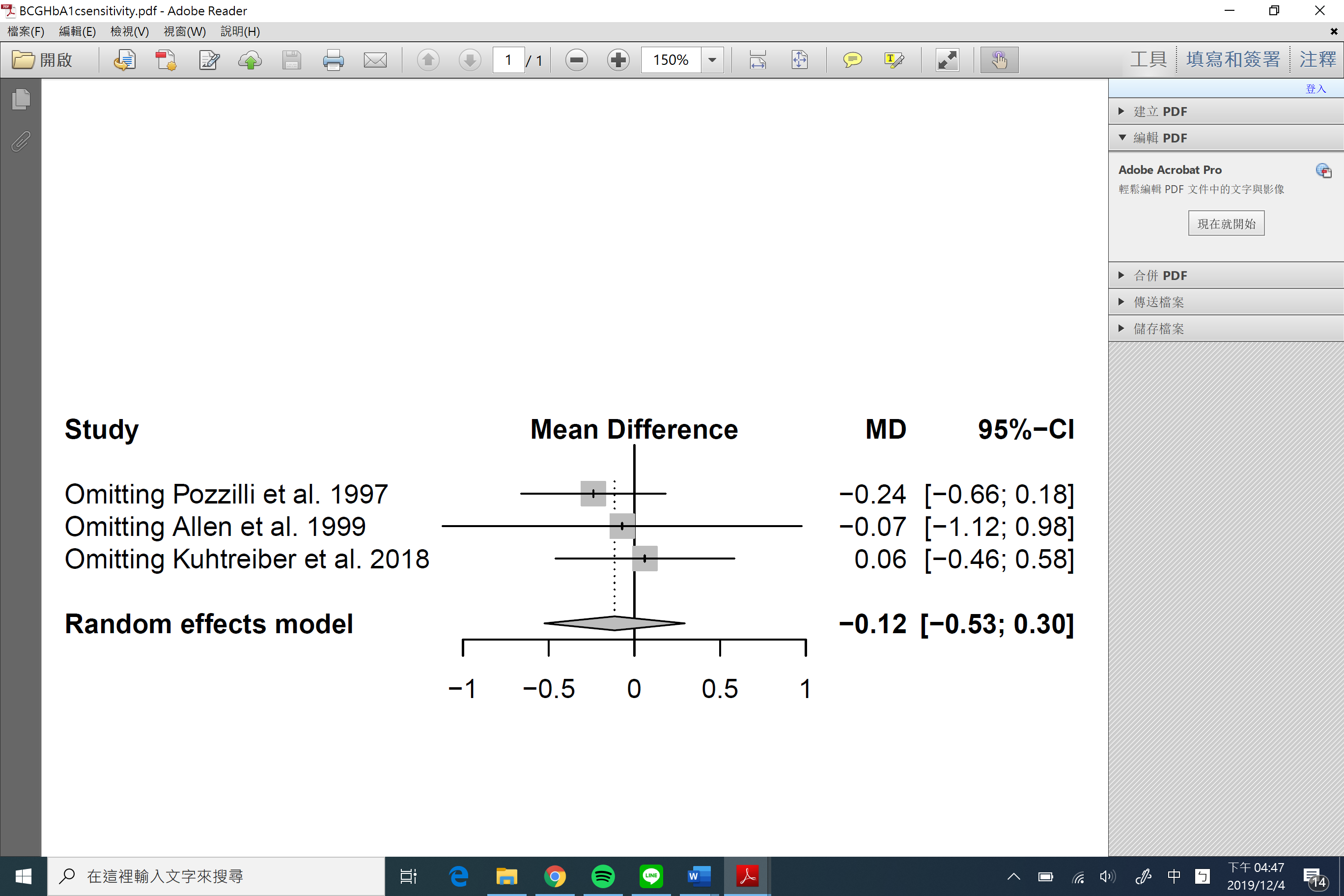
**Figure S1. Sensitivity analyses of BCG on pooled HbA1c level (%) in T1DM patients by omitting each study**

BCG, Bacillus Calmette–Guerin; HbA1c, glycated hemoglobin; MD, mean difference; CI, confidence interval
